# Bayesian and maximum likelihood phylogenetic analyses of protein sequence data under relative branch-length differences and model violation

**DOI:** 10.1186/1471-2148-5-8

**Published:** 2005-01-28

**Authors:** Jessica C Mar, Timothy J Harlow, Mark A Ragan

**Affiliations:** 1 Department of Mathematics, The University of Queensland, Brisbane, Qld 4072, Australia; 2 Department of Biostatistics, Harvard School of Public Health, Boston, MA 02115 USA; 3 Institute for Molecular Bioscience, The University of Queensland, Brisbane, Qld 4072, Australia; 4 Australian Research Council (ARC) Centre in Bioinformatics, Australia; 5 Program in Evolutionary Biology, Canadian Institute for Advanced Research, Canada

## Abstract

**Background:**

Bayesian phylogenetic inference holds promise as an alternative to maximum likelihood, particularly for large molecular-sequence data sets. We have investigated the performance of Bayesian inference with empirical and simulated protein-sequence data under conditions of relative branch-length differences and model violation.

**Results:**

With empirical protein-sequence data, Bayesian posterior probabilities provide more-generous estimates of subtree reliability than does the nonparametric bootstrap combined with maximum likelihood inference, reaching 100% posterior probability at bootstrap proportions around 80%. With simulated 7-taxon protein-sequence datasets, Bayesian posterior probabilities are somewhat more generous than bootstrap proportions, but do not saturate. Compared with likelihood, Bayesian phylogenetic inference can be as or more robust to relative branch-length differences for datasets of this size, particularly when among-sites rate variation is modeled using a gamma distribution. When the (known) correct model was used to infer trees, Bayesian inference recovered the (known) correct tree in 100% of instances in which one or two branches were up to 20-fold longer than the others. At ratios more extreme than 20-fold, topological accuracy of reconstruction degraded only slowly when only one branch was of relatively greater length, but more rapidly when there were two such branches. Under an incorrect model of sequence change, inaccurate trees were sometimes observed at less extreme branch-length ratios, and (particularly for trees with single long branches) such trees tended to be more inaccurate. The effect of model violation on accuracy of reconstruction for trees with two long branches was more variable, but gamma-corrected Bayesian inference nonetheless yielded more-accurate trees than did either maximum likelihood or uncorrected Bayesian inference across the range of conditions we examined. Assuming an exponential Bayesian prior on branch lengths did not improve, and under certain extreme conditions significantly diminished, performance. The two topology-comparison metrics we employed, edit distance and Robinson-Foulds symmetric distance, yielded different but highly complementary measures of performance.

**Conclusions:**

Our results demonstrate that Bayesian inference can be relatively robust against biologically reasonable levels of relative branch-length differences and model violation, and thus may provide a promising alternative to maximum likelihood for inference of phylogenetic trees from protein-sequence data.

## Background

The inference of phylogenies from molecular sequence data, like most other quantitative problems in science, is most powerful within a model-based statistical framework. Sophisticated models are available to describe how sequences change along branches of a tree, and how the rate of sequence change varies among sites. Statistical measures describe both the quality of inferred trees, and the confidence that can be assigned to the existence and position of subtrees. Likelihood-based approaches have proven especially powerful for inferring phylogenetic trees [1,2] but are computationally expensive owing both to the form of the likelihood function itself, and to the need to search the multidimensional space of possible outcomes (tree space) for optimal trees. This computation then must be repeated, typically 100–1000 times, if the nonparametric bootstrap [3] is used to estimate the support for specific subtrees. As a result, maximum-likelihood inference can be prohibitively slow for problems that involve large numbers of aligned sequences, comprehensive search of tree space, and/or many bootstrap replicates. The much faster RELL approximation [4,5] can in principle replace the bootstrap, although so far it has not been extensively investigated with large datasets [6].

At the same time, the ongoing success of genomic sequencing – new microbial genome sequences are now appearing at the rate of at least one per week – is yielding a wealth of ever-larger gene and protein datasets suitable for large-scale analysis of deep issues in comparative and evolutionary genomics, *e.g. *the relative contributions of vertical and lateral gene transfer to genomic diversity [7,8]. However, these datasets are too numerous, and many of them too large, for ready analysis by likelihood inference. For example, using an automated phylogenetics pipeline [9] we have generated more than 22400 protein datasets having up to 144 sequences each, for which we must infer trees. There is consequently much interest in approaches that offer improved search efficiencies while remaining within a model-based statistical framework.

Among the most interesting of these is Bayesian inference, in which the posterior probability of a hypothesis (*i.e. *a tree) is associated with its probability of being correct, given the prior probability, model and data [2,10]. Although posterior probabilities cannot be computed analytically for interestingly large datasets, Markov chain Monte Carlo (MCMC) methods can be used to find and examine equilibrium distributions of trees, on the basis of which we can make probability statements about the true tree [10-14]. Bayesian inference of phylogeny supports sophisticated evolutionary models, while MCMC, particularly with heated chains (Metropolis-coupled MCMC), recovers from the posterior probability distribution a sample of topologies within which the empirical relative frequency of a given topology converges to its corresponding marginal posterior probability [15]. The topology with highest relative frequency in this sample is typically reported, and posterior probabilities of subtrees can be estimated by consensus from the topologies visited [10,13].

Bayesian phylogenetic inference has been applied to simulated [16-18] as well as empirical nucleotide datasets (see below). The results establish the applicability and computational efficiency of the Bayesian MCMC approach to molecular phylogenetic inference. However, concerns have arisen about (1) finding optimal trees, (2) overly liberal confidence estimates on subtrees [19-24], and (3) the possibility that Bayesian inference can resolve topological features (*e.g. *internal edges, hence subtrees) that do not actually exist [16]. Certain other issues have not been systematically addressed with nucleotide data, notably the robustness of Bayesian inference to relative branch-length differences and to model violation.

Much less is known about the behaviour of Bayesian inference with protein-sequence data. While there is no *a priori *reason that protein-sequence data should be more or less problematic than nucleotide data for Bayesian phylogenetics, gene and protein sequences have distinct statistical properties, and are subject to different selective constraints; so it is not inconceivable that, in practice, the corresponding models of sequence change might tend to fail in different ways, or to different extents. Bayesian inference has been applied to inference of phylogenetic trees for cytochrome *b *[25], elongation factor 1α [26], hydroperoxidases [27], 3-hydroxy-3-methylglutaryl coenzyme A reductase (HMGR) [18], membrane-intrinsic protein [28], and concatenated mitochondrial protein [29] and larger [30] datasets. Douady *et al. *[18] report a linear, if noisy, correlation between bootstrap proportion and Bayesian posterior probability for a 15-taxon HMGR protein dataset. As in the case of nucleotide data, the robustness of Bayesian inference to branch-length differences and model violation with protein-sequence datasets remains unexplored.

To better characterize the behavior of Bayesian phylogenetic inference with protein-sequence data, we have applied MrBayes [31,32] to both empirical and simulated data. Based on the analysis of 21 empirical protein datasets, we compare maximum likelihood bootstrap proportion and Bayesian posterior probability as estimates of subtree confidence. From analyses of simulated data known to contain phylogenetic signal, we address the fidelity with which the correct topology is recovered under progressively extreme ratios of branch-length differences, both under the correct model of sequence change (the model under which the data were evolved) and under a model that incorporates different amino acid substitution probabilities. Given our ongoing research on lateral gene transfer (above), we were particularly interested in the number of discrete events (edits: [33]) separating inferred from known trees.

In this work we compare and contrast results obtained using two popular software programs, PROML [34] and MrBayes [31], as well-developed implementations of the ML and Bayesian approaches to phylogenetic inference respectively. Although the comparison is illustrative, it would be an oversimplification to view these two approaches as diametric opposites, or even as fundamentally mutually exclusive. Both likelihood and Bayesian are general statistical frameworks, with high-level decision criteria (the Akaike Information Criterion, or AIC [35] and Bayesian Information Criterion, or BIC [36], respectively: see also [37]) and associated apparatus for *e.g. *examining solution space, estimating support, and assessing stability to stochastic error. Only a subset of these broad bodies of theory and practice has so far been applied to phylogenetic inference, and even less implemented in platform-independent software. If we apply BIC to alternative trees and assume equal prior probabilities, it becomes possible to estimate Bayesian posteriors from their likelihood differences, linking the two approaches at this level [6,38]. Stochastic approaches related to MCMC, including simulated annealing [39] and the generalised Gibbs sampler [40], can be used to search tree space in ML. The nonparametric bootstrap, more typically applied in conjunction with parsimony and ML, has proven useful in assessing subtree support in Bayesian inference [6,18]. The application of likelihood in hybrid methods [41-43], the likelihood ratchet [44], and a metapopulation genetic algorithm [45] lie farther beyond the scope of this discussion, but illustrates the potential for further development of both of these phylogenetic approaches beyond the specific implementations used in this study.

## Results

### Empirical data

#### Topology

We inferred maximum likelihood (ML) and Bayesian (B) trees for the 21 empirical protein-sequence datasets. For 7 of these datasets, every combination of approach and model that we investigated (ML-JTT-HMM, ML-JTT-gamma, B-JTT, B-EQ: see *Empirical data *under Methods) yielded the same topology. Interestingly, for these, the bootstrap consensus ML trees were topologically identical to the ML and Bayesian trees, indicating that the sequences in these datasets show a high degree of internal consistency across positions (*i.e. *bear few homoplasies). For another 10 datasets, one or more of these four approaches yielded a tree that differs slightly (edit distance ≤ 2) from the others. No pattern was obvious among these disagreements: the differences do not, for example, systematically separate ML from Bayesian trees. For these 10 datasets, the differences are simple edits, *e.g. *-(A(BC)) to -(B(AC)), or -((AB)(CD)) to -(A(B(CD))). For the remaining 4 datasets, one or more of the four approaches yielded a tree that differed more substantially (edit distance ≥ 3). Over these examples, the datasets that yield more-conflicted trees are slightly larger (mean, 12.25 sequences each) than those yielding slightly conflicted (mean, 11.50 sequences each) or identical trees (mean, 10.86 sequences each), although the numbers of datasets involved are too few for this observation to be generalized.

#### Support for subtrees

**Figure 1:**
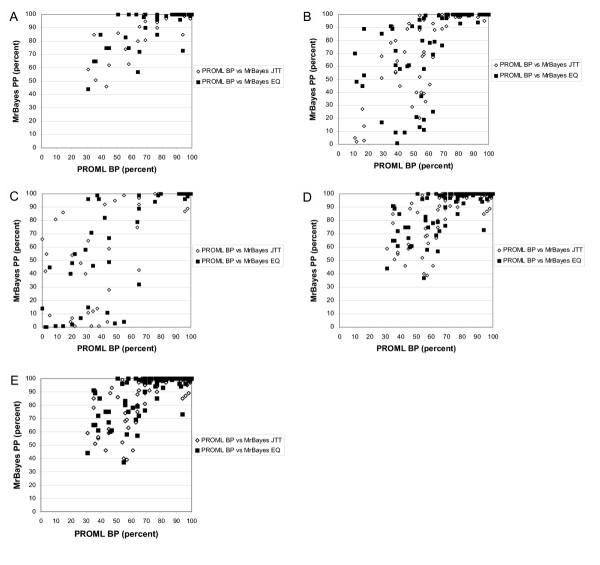
**Empirical data: relationship between ML consensus bootstrap proportion and Bayesian posterior probability. **Comparison of PROML bootstrap proportions (horizontal axes) with Bayesian posterior probabilities (vertical axes) for all internal nodes in trees inferred from 21 empirical protein-sequence datasets. Data are for trees inferred by gamma-corrected ML under JTT, *versus *those inferred by gamma-corrected Bayesian inference under JTT (open diamonds) or under EQ (closed squares), (A) for the 7 datasets for which the two ML and two Bayesian trees (see text) are topologically identical, (B) for the 10 datasets for which at least one ML or Bayesian tree (see text) differs slightly (edit distance ≤ 2) from the other three, (C) for the 4 datasets for which at least one tree differs more substantially (edit distance ≥ 3), (D) for the subset of internal nodes, within the latter 14 non-identical trees, that subtend identical subtrees, and (E) for data in panels (A) and (D) plotted together.

We compared PROML bootstrap proportions (BPs) with Bayesian posterior probabilities (PPs) separately for all subtrees among the three groups of trees inferred from these 21 empirical datasets: the 7 trees for which all four sets of approaches and models (ML-JTT-HMM, ML-JTT-gamma, B-JTT, B-EQ) yielded the same topology, the 10 for which one or more approach yielded a slightly different tree, and the 4 for which one or more tree differed more substantially. In Figure 1, we show the relationship between BP (from PROML using the 8-category gamma distribution: see Methods) and PP for subtrees in these three groups of trees; results for PROML using the hidden Markov model (HMM) are very similar (results not shown). Where ML and Bayesian approaches yield the same topology, the relationship between BP and PP can most simply be fit by a straight line (P-values for linearity are between e-10 and e-13 depending on the subset of data examined). With very few exceptions, however, the PP values are greater, and almost all of the BP values above 80% correspond to 100% PP (Figure 1, panel A). For the 14 datasets for which at least one of the four approaches yields a conflicting tree, the relationship between BP and PP appears much more complex (Figure 1, panels B,C), although for the subset of non-conflicting subtrees among these 14 datasets (Figure 1, panel D) the relationship between BP and PP is similar to that for topologically identical trees (Figure 1, panel A). Panel E combines data for all non-conflicting subtrees (panels A and D). In all of these views on the data (Figure 1, panels A-E), however, most points lie above and to the left of the diagonal (Table 1), indicating that for empirical protein-sequence datasets, as for DNA-sequence datasets (see Introduction), Bayesian PPs tend to be more generous than nonparametric BPs as estimates of confidence in subtrees.

**Table 1:** Linear fit equations for data in Figure 1. Slope, y-intercept, significance, and R^2 ^values for linear equations relating bootstrap proportion and Bayesian posterior values shown in panels A, D and E of Figure 1, *i.e. *for all nodes subtending identical subtrees among the 21 empirical protein-sequence datasets, regardless of whether the corresponding full ML and Bayesian trees are topologically identical or not.

**Data**^1^	**Panel**	**Slope**	**SE^2^**	**Signif**	***y*-Intcpt**	**SE**	**Signif**	**Mult R^2^**	**Adj R^2^**
JTT model, all data	A	0.4993	0.0536	0.001	51.823	4.568	0.001	0.6207	0.6136
	D	0.5150	0.0398	0.001	50.125	3.441	0.001	0.5279	0.5247
	E	0.5101	0.0322	0.001	50.625	2.773	0.001	0.5508	0.5486
EQ model, all data	A	0.4557	0.0572	0.001	55.661	4.871	0.001	0.5452	0.5367
	D	0.4517	0.0352	0.001	56.269	3.050	0.001	0.5227	0.5195
	E	0.4531	0.0298	0.001	56.077	2.569	0.001	0.5297	0.5274
JTT model, BP <85%	A	0.7536	0.1771	0.001	38.180	10.819	0.01	0.4880	0.4610
	D	0.7816	0.1258	0.001	34.931	8.115	0.001	0.4081	0.3976
	E	0.7694	0.1020	0.001	36.112	6.487	0.001	0.4251	0.4176
EQ model, BP <85%	A	0.6909	0.1809	0.01	43.124	11.054	0.001	0.4342	0.4044
	D	0.6837	0.1099	0.001	43.086	7.089	0.001	0.4088	0.3982
	E	0.6843	0.0922	0.001	43.174	5.866	0.001	0.4170	0.4095

^1^All data: values based on all data shown in the respective panel in Figure 1; BP <85%: values based on only those data for which the value of the PROML bootstrap proportion is less than 85%.

^2^SE, standard error; Signif, significance (probability level that estimate >|t|); *y*-Intcpt, *y*-intercept; Mult R^2^, multiple R^2^; Adj R^2^, adjusted R^2^.

From our data, it is not possible to reject the hypothesis that the relationship between BP and PP has the same slope whether the Bayesian inference is conducted using JTT, or EQ, as the model of sequence change. Analysis of covariation (ANCOVA) yields probabilities 0.579 (Panel A), 0.235 (Panel D) and 0.195 (Panel E) that the lines described in Table 1 differ in slope between the JTT and EQ models. When data having >85% BP are removed from analysis, the probabilities become 0.806, 0.559 and 0.537 respectively, but equivalence still cannot be rejected. Given the limitations of these data, we did not attempt a more-complete analysis, *e.g. *involving minority subtrees (those not in the extended 50% majority-rule consensus) or higher-order (sigmoidal) fit curves.

Because for these trees the true molecular phylogeny is unknown, these results do not speak to the accuracy of the inferred topologies. For this, it is necessary to examine inferences based data simulated on trees of known topology.

### Simulated data

#### Topology

**Figure 2:**
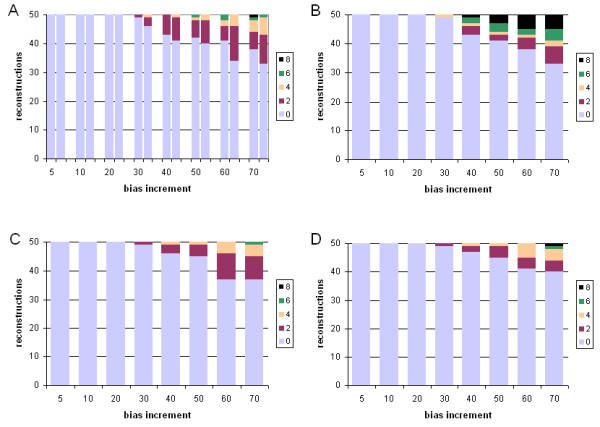
**Comparative performance with simulated data: correct model, single long branch, symmetric distance **Performance at different branch-length ratios of ML and Bayesian inference with simulated protein-sequence data evolved on a tree having a single long branch, measured as Robinson-Foulds symmetric distance. The JTT model was used for both sequence evolution and tree inference. Number (out of 50) of accurately reconstructed topologies (vertical axes) *versus *branch-length ratio (horizontal axes), where inference was by (A) gamma-corrected PROML, (B) Bayesian uncorrected for ASRV, with uniform prior, (C) gamma-corrected Bayesian with uniform prior, and (D) gamma-corrected Bayesian with exponential prior. Shading codes for each different distance are shown in the small box at the right of each panel (A-D). Thus the right-hand bar in panel B shows that using Bayesian inference uncorrected for ASRV and assuming a uniform prior, with a dataset generated on a tree in which one branch was lengthened 70-fold, 33 of 50 independent trees recovered the correct topology (Robinson-Foulds symmetric distance zero); 6 differed topologically in ways that involved a single node (distance two); 2 differed in ways that involved two adjacent nodes (distance four); 4 were at distance six; and the remaining 5 were at the maximum symmetric distance, eight. See text for explanation of dual bars in Panel A.

**Figure 3:**
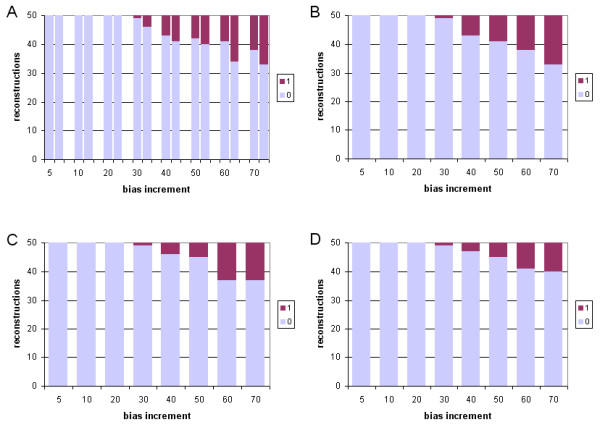
**Comparative performance with simulated data: correct model, single long branch, edit distance. **Performance at different branch-length ratios of ML and Bayesian inference with simulated protein-sequence data evolved on a tree having a single long branch, measured as edit distance. The JTT model was used for both sequence evolution and tree inference. Models, panels and axes are as in Figure 2.

We first examine cases where tree inference was carried out under the same model (JTT) as that used to generate the data, and where a single branch was progressively extended in length (see Methods). When trees were inferred using gamma-corrected ML, the correct tree was recovered in 100% (50/50) of the cases in which the relative branch-length difference was 5-, 10- or 20-fold (Robinson-Foulds symmetric distance in Figure 2, panel A, and edit distance in Figure 3, panel A). The frequency of inaccurately reconstructed trees increased with further increase in relative branch-length difference, and the inaccurately reconstructed trees were increasingly inaccurate as judged by Robinson-Foulds symmetric distance (which measures the number of bipartitions involved in topological incongruence) although not by edit distance (which measures the number of break-and-reanneal differences without reference to bipartitions).

We investigated two ways of assessing the performance of ML inference. In panel A of Figures 2 and 3, a pair of bars is shown at each value of branch-length difference. The left-hand bar shows performance assessed over 50 single ML reconstructions (one from each of the 50 datasets evolved at that relative length increment), while the right-hand bar shows performance assessed over 50 consensus trees (each of which summarizes 10 bootstrap replicates for each of the same 50 datasets). For datasets having a single long branch, the two representations yield very similar results, with the individual ML results usually showing slightly better performance. By contrast, the situation was reversed for datasets with two long branches. Although consensus is an appropriate way to summarize bootstrap results, nonparametric bootstrap proportions do not measure support for subtrees in a simple, direct and unbiased manner [46-48]. For this reason, one might question whether an approach based on bootstrap and consensus appropriately summarizes the performance of ML for comparison with Bayesian inference, as Bayesian posteriors *do *directly measure subtree probabilities (given the priors, model and data). The similarities we observe in both magnitude and trend for the two approaches demonstrate that the comparison we are making between ML and Bayesian inference does not, in these cases at least, depend on whether or not the performance of ML is assessed using an approach that involves the nonparametric bootstrap.

With Bayesian inference, inaccurately reconstructed trees were also first seen at the 30-fold branch-length ratio (Figure 2, panels B-D, and Figure 3, panels B-D). Compared with the ML consensus result (panel A, right-hand bar), Bayesian inference almost always yielded a higher frequency of accurate reconstructions. However, unless correction was made for ASRV, the inaccurate trees, although fewer in number, could be more inaccurate as judged by symmetric distance. Gamma correction for ASRV greatly reduced the frequency of the most inaccurate reconstructions, yielding results (Figure 2, panels C,D) noticeably better than with gamma-corrected ML. In our simulations, use of an exponential prior (Figure 2, panel D) gave slightly fewer inaccurate trees at the most-extreme branch-length ratios, although the difference is not statistically significant (Wilcoxon matched-pairs signed-rank test).

**Figure 4:**
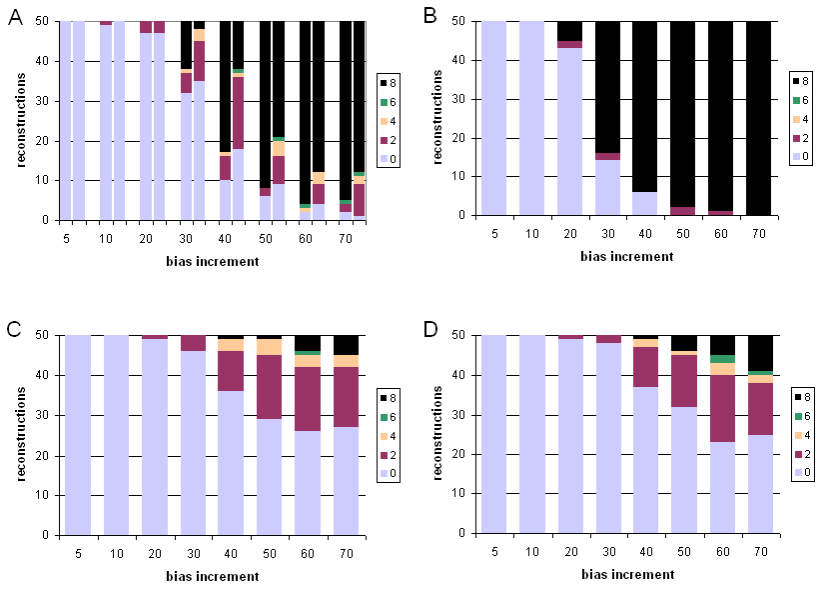
**Comparative performance with simulated data: correct model, two long branches, symmetric distance. **Performance at different branch-length ratios of ML and Bayesian inference with simulated protein-sequence data evolved on a tree having two long branches, measured as Robinson-Foulds symmetric distance. The JTT model was used for both sequence evolution and tree inference. Models, panels and axes are as in Figure 2.

**Figure 5:**
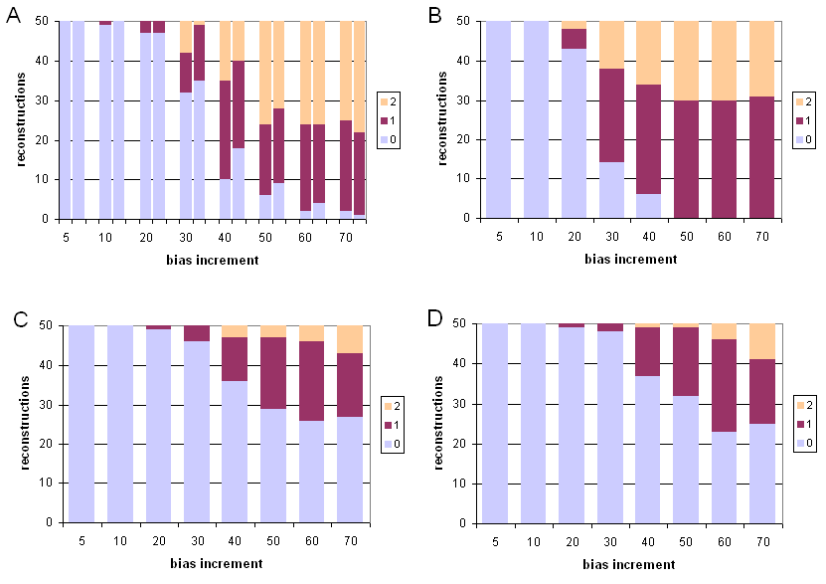
**Comparative performance with simulated data: correct model, two long branches, edit distance. **Performance at different branch-length ratios of ML and Bayesian inference with simulated protein-sequence data evolved on a tree having two long branches, measured as edit distance. The JTT model was used for both sequence evolution and tree inference. Models, panels and axes are as in Figure 2.

In Figures 4 and 5 we present the results of tree inference carried out under the same model (JTT) as that used to generate the data, but where two branches were progressively extended relative to the others. For each of the four sets of approaches and models considered, the first inaccurate tree reconstruction was observed at 20-fold relative difference. At higher branch-length ratios, relative performance among the four suites of approaches and models is much more striking than was seen when only a single long branch was present. With gamma-corrected ML, for example, by 50-fold ratio only 9/50 tree topologies are accurately inferred, and at 70-fold ratio only 1/50 (Figure 4, panel A). ASRV-uncorrected Bayesian inference (Figure 4, panel B) performs even worse, with no accurate inferences at branch-length ratios 50 or greater. However, gamma correction (Figure 4, panels C,D) yielded a much higher frequency of accurate reconstructions, with the uniform prior performing better than the exponential prior at the more extreme ratios (Wilcoxon P ≤ 0.003906 at 70-fold) as judged by symmetric distance. About three-quarters (74.9%) of the inaccurate topologies inferred in the case of two differentially lengthened branches showed long-branch attraction, *i.e. *the long branches were topologically adjacent in the reconstructed tree.

#### Support for subtrees

**Figure 6:**
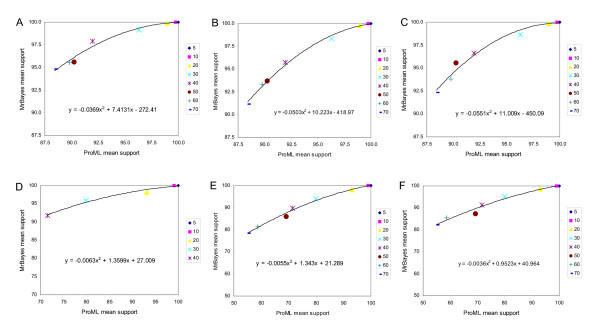
**Simulated data: relationship between ML consensus bootstrap proportion and Bayesian posterior probability. **Relationship between bootstrap proportion for ML consensus trees, and posterior probability for Bayesian trees, for datasets with one (A-C) or two (D-F) branches of relatively greater length. Bayesian trees were inferred (A and D) without ASRV correction and with a uniform prior, (B and E) with gamma correction for ASRV and with a uniform prior, and (C and F) with gamma correction and with an exponential prior. Panel D does not show data at relative branch-length ratios ≥ 50 because none of the trees inferred at these branch-length ratios recovered the known topology.

In Figure 6 we compare the quantitative support for subtrees, in trees inferred from these simulated datasets by ML and Bayesian approaches, as assessed by bootstrap proportion and posterior probability respectively. Panels A-C show the comparisons based on 1600 extended majority-rule consensus trees for datasets with one long branch (50 ML trees at each of eight branch-length ratios, compared with 50 Bayesian trees at each of the same ratios, over three combinations of ASRV correction and prior probability distribution), and panels D-F are based on 1600 consensus trees for datasets with two long branches. The values shown were derived by summation of BP, and of PP, values over all internal nodes only for the trees that were accurately inferred (*i.e. *identical with the known topology). By structuring the comparison in this way, we avoid cases where the ML consensus might be topologically different from the best component tree, and avoid dealing with the plethora of cases and sub-cases that arise in comparing topologically non-congruent trees.

For all three combinations of ASRV correction and prior (corresponding to panels B-D of Figures 2, 3, 4, 5), the relationship between BP and PP, structured in this way, is best fit by a smooth curve that reaches 100% PP only at BP 99.75% (Figure 6, panels A-D) or BP 100% (panels E-F), *i.e. *shows little or no "saturation". For both the single- and two-long-branches cases, the PP is greatest, compared to BP, for Bayesian trees inferred without correction for ASRV, and least generous for gamma-corrected trees where the prior distribution was assumed to be uniform. Unsurprisingly, the lower values of subtree support, as measured both by BP and by PP, arise from the trees with the most extreme relative branch length differences.

#### Performance under model violation

**Figure 7:**
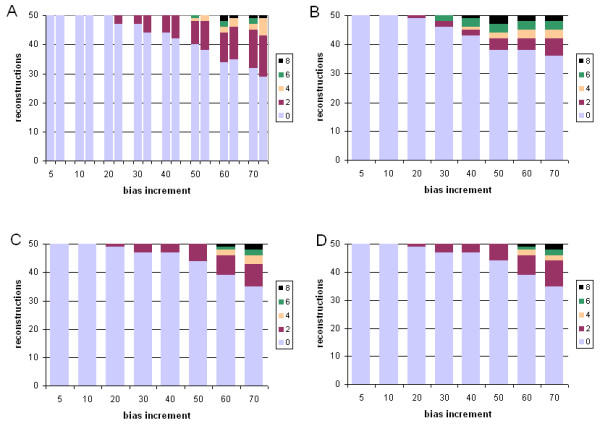
**Comparative performance with simulated data: incorrect model, one long branch, symmetric distance. **Performance at different branch-length ratios of ML and Bayesian inference with simulated protein-sequence data evolved on a tree having a single long branch, measured as Robinson-Foulds symmetric distance. Data were evolved under the mtmam model, but trees were inferred under the JTT model. Panels and axes are as in Figure 2.

**Figure 8:**
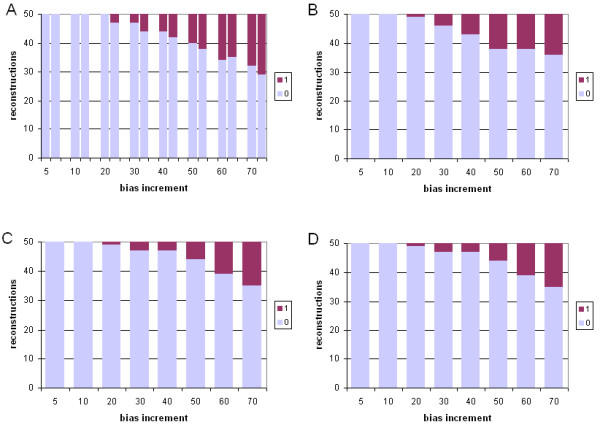
**Comparative performance with simulated data: incorrect model, one long branch, edit distance. **Performance at different branch-length ratios of ML and Bayesian inference with simulated protein-sequence data evolved on a tree having a single long branch, measured as edit distance. Data were evolved under the mtmam model, but trees were inferred under the JTT model. Models, panels and axes are as in Figure 2.

**Figure 9:**
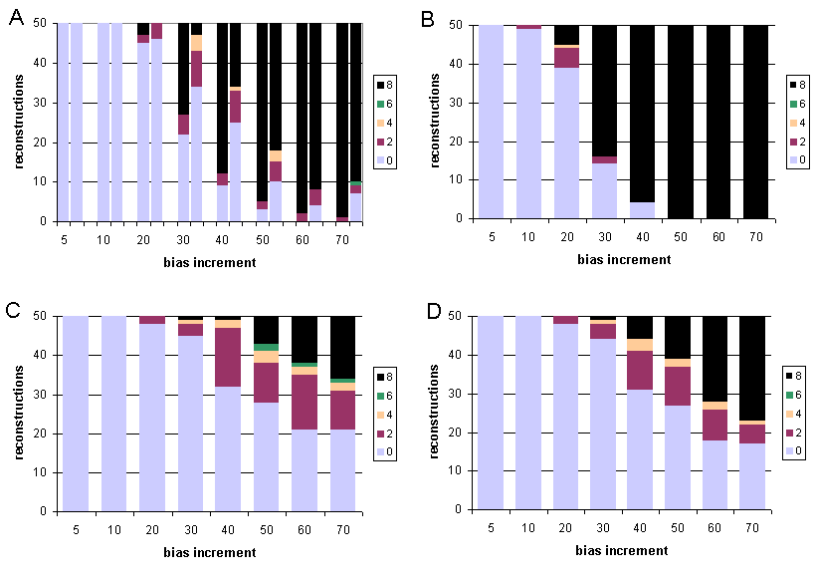
**Comparative performance with simulated data: incorrect model, two long branches, symmetric distance. **Performance at different branch-length ratios of ML and Bayesian inference with simulated protein-sequence data evolved on a tree having two long branches, measured as Robinson-Foulds symmetric distance. Data were evolved under the mtmam model, but trees were inferred under the JTT model. Models, panels and axes are as in Figure 2.

**Figure 10:**
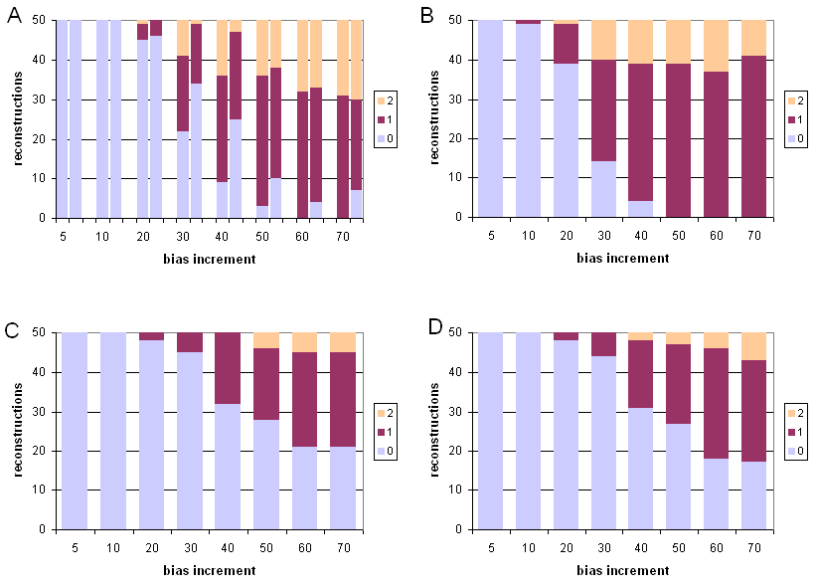
**Comparative performance with simulated data: incorrect model, two long branches, edit distance. **Performance at different branch-length ratios of ML and Bayesian inference with simulated protein-sequence data evolved on a tree having two long branches, measured as edit distance. Data were evolved under the mtmam model, but trees were inferred under the JTT model. Models, panels and axes are as in Figure 2.

We next compared the performance of ML and Bayesian inference under violation of the model of sequence change, by evolving datasets under a mammalian mitochondrial model (mtmam), but inferring trees under the JTT model (see Methods). Performance of each of the four sets of approaches and methods was assessed by comparing four measures: the branch-length ratio at which inaccurate trees were first observed; the total number of steps (summed over the eight ratios) by which the 400 trees differ from the true topology; the weighted sum ("burden") of these steps; and the mean number of steps by which each inaccurate tree differs from the known tree. The latter two measures were each calculated using both Robinson-Foulds symmetric distance, and edit distance, yielding six comparisons in all. A more-complete description is provided at footnote 2 of Table 2. Performance in the case of one long branch is summarized in Figures 7 and 8, and in the case of two long branches in Figures 9 and 10. In Table 2 we summarize and compare the performance of ML and Bayesian inference with these datasets under the correct, and an incorrect, model.

**Table 2:** Simulated data: comparative performance under correct and incorrect models. Performance of maximum-likelihood and Bayesian phylogenetic inference without, and with, violation of the model of protein sequence change, for trees with one, or two, relatively long branches.

**One long branch**												
	***No model violation^1^***						***Model violation^1^***					

	First^2^	Wrong	Burd SD	Mean SD	Burd ED	Mean ED	First	Wrong	Burd SD	Mean SD	Burd ED	Mean ED

ML^3^	30	56	144	2.57	56	1.00	20	65	164	2.52	65	1.00
BUU	30	46	232	5.04	46	1.00	20	50	222	4.44	50	1.00
BGU	30	36	96	2.67	36	1.00	20	39	118	3.03	39	1.00
BGE	30	28	88	3.14	28	1.00	20	39	116	2.97	39	1.00

**Two long branches**												

	***No model violation***						***Model violation***					

	First	Wrong	Burd SD	Mean SD	Burd ED	Mean ED	First	Wrong	Burd SD	Mean SD	Burd ED	Mean ED

ML	20	186	1124	6.04	273	1.47	20	174	1166	6.70	207	1.19
BUU	20	237	1854	7.82	326	1.38	10	244	1900	7.79	299	1.23
BGU	20	87	270	3.10	104	1.20	20	105	468	4.46	119	1.13
BGE	20	86	314	3.65	101	1.17	20	115	650	5.65	131	1.14

^1 ^Protein-sequence data were evolved under the Jones *et al. *(JTT) or, alternatively, mammalian mitochondrial (mtmam) model of sequence change, and trees were inferred assuming the JTT model.

^2 ^Performance was measured by six indices: *First*, the lowest investigated branch-length ratio at which at least one inaccurately reconstructed tree was found; *Wrong*, the number of inaccurately inferred trees out 400 (8 branch-length ratios × 50 replicates at each ratio); *BurdSD*, the bipartition burden, calculated as the Robinson-Foulds symmetric distance by which each tree differs from the known tree, summed over the 400 trees; *MeanSD*, the mean Robinson-Foulds symmetric distance per inaccurate tree; *BurdED*, the edit burden, calculated as the edit distance by which each tree differs from the known tree, summed over the 400 trees; and *MeanED*, the mean edit distance per inaccurate tree.

^3 ^Inference methods: *ML*, protein maximum likelihood with gamma ASRV correction; *BUU*, Bayesian inference, uncorrected for ASRV, uniform prior; *BGU*, Bayesian inference, gamma ASRV correction, uniform prior; and *BGE*, Bayesian inference, gamma ASRV correction, exponential prior. See text for further details.

For datasets in which a single branch was of relatively greater length, violating the model of sequence change degraded performance of the four approaches (Table 2). In each case, inaccurate trees were first observed at 20-fold branch-length ratio, earlier than the 30-fold ratio seen in the absence of model violation. Inaccurate trees were more numerous, in comparison with inference under the correct model. With ML, each inaccurate tree was about as inaccurate under the incorrect model as under the correct one, as measured by symmetric distance (Table 2). With Bayesian inference, inaccurate trees produced under the wrong model were, unexpectedly, sometimes less inaccurate than those inferred under the correct model (Table 2), and in one case (no correction for ASRV, uniform prior distribution) the total burden of changes was less, as measured by symmetric distance. Exclusion of results from the 70-fold data (results not shown) demonstrated that this effect is not due to a loss of dynamic range at extreme values.

For datasets containing two long branches, model violation affected performance of ML and Bayesian inference differently. With ML, inference under the wrong model produced a somewhat lower frequency of topologically inaccurate trees, although each inaccurate tree was more inaccurate as judged by symmetric distance (Table 2). With Bayesian inference, use of the wrong model increased the frequency of inaccurate trees, and each inaccurate tree tended to be more inaccurate as measured by symmetric distance. With Bayesian inference uncorrected for ASRV and using a uniform prior, the first inaccurate tree appeared at a ratio of only 10, and no accurate trees were recovered at ratios 50 or higher; although by most indices the performance was not further degraded by violation of the model of sequence change, performance was already quite poor, and not much dynamic range remained available. Use of an exponential prior again made a significant difference only with two long branches and assessment using Robinson-Foulds symmetric distance (Wilcoxon P ≤ 0.00097 and P ≤ 0.00003 for degraded performance at 60- and 70-fold branch-length ratios respectively).

## Discussion

Unlike the situation with established approaches based on pairwise distances, parsimony or maximum likelihood, relatively little experience has accumulated so far on the application of Bayesian approaches to phylogenetic inference, especially for protein-sequence datasets. In this work we (a) extend the comparison of Bayesian posterior probabilities with nonparametric bootstrap proportions as measures of confidence in subtrees, (b) systematically investigate the robustness of ML and Bayesian inference to branch-length differences, and (c) compare the behavior of these two approaches to one specific violation of the model of sequence change. We used two measures to compare topologies (Robinson-Foulds symmetric distance, and edit distance), and it is clear that they captured different facets of topological incongruence.

### Support for subtrees

Using 21 empirical protein-sequence datasets, we compared Bayesian posterior probabilities with bootstrap proportions based on ML as measures of support for subtrees. To make this comparison as fair as possible, we restricted our analysis to a model of sequence change (JTT) and a correction for ASRV (discrete approximation to the gamma distribution) available in both PROML and MrBayes. We did not optimize models separately for each approach or for each dataset, as JTT+gamma represents the most-parameterized combination that these two programs support in common. It is therefore possible that some of the difference observed between the two measures results from differential sensitivity of ML and Bayesian inference, as implemented in these programs, to deviation of JTT and the discrete gamma distribution from an optimal description of the processes of sequence change that actually gave rise to these sequences (but see the final paragraph under *Model violation*, below). As it is unlikely that any existing model – certainly any that fails to account for lineage-specific processes and temporal variations along these lineages – fully represents the historical complexity of molecular evolution, the same criticism could be levelled, albeit perhaps in lesser degree, against all current applications of statistically based phylogenetic inference to empirical datasets.

The data presented in Figure 1 demonstrate that, at least for these protein-sequence datasets, Bayesian PPs tend to offer a more-generous estimate of subtree reliability than does the nonparametric bootstrap combined with ML. This result supports and extends previous studies with DNA- [16-18,20-22,49] and protein-sequence data [18,19]. Bayesian PPs and nonparametric bootstrap BPs are not commensurate [17,48] and may be seen as "potential upper and lower bounds of node reliability" respectively (page 248 of [18]). (Being more-generous than a too-conservative measure does not, of course, imply that Bayesian PPs must be too-generous.) Our results strongly suggest that the interpretation of BPs and PPs being developed for nucleotide sequences will be applicable, as well, to protein sequences.

For sets of consensus trees inferred from simulated protein-sequence data (Figure 6), Bayesian PPs tend to be more generous than nonparametric BPs in estimates of subtree support. However, whereas for empirical protein-sequence data (and nucleotide-sequence data: see references cited immediately above) PPs tend to "saturate", *i.e. *reach 100% at BP values less than 100% (here around 80%), with our simulated data the relationship between BP and PP resembles a smooth curve reaching 100% PP only at BP greater than 99%. Further studies will be required to disentangle why little or no saturation was observed; possibilities include the structure of our simulated trees (*e.g. *their symmetry, or an usually regular spacing of internal nodes), the way that data were evolved on these trees (*e.g. *assuming strict independence among sites, or rigorous adherence to the JTT model), and/or the way we summarize the support data for ML (*via *extended majority-rule consensus trees).

### Relative branch-length differences

Dissimilar sequences (represented in phylogenetic trees as long branches) create difficulties in phylogenetic analysis. The issue has been most extensively explored in parsimony analysis, where branch length can be an important consideration, *e.g. *in selection of outgroups and resolution of topologically problematic regions. Parsimony analysis is particularly susceptible to "long branch attraction" (LBA) artefacts, in which two or more branches are resolved adjacent in a tree solely because they are highly divergent from the others [2]. ML inference can be more robust against LBA, although to our knowledge this has been not been specifically examined for protein-sequence data. We are unaware of any systematic examination of the degree to which Bayesian phylogenetic inference is robust against branch length-based artefact.

Our results (Figures 2, 3, 4, 5) indicate that for protein-sequence datasets of this size, both gamma-corrected ML and Bayesian inference can be robust to artefact arising from the levels of dissimilarity likely to be encountered in empirical biological data. Both ML and Bayesian inference can be fully robust (within our limits of detection) to at least a 20-fold relative length ratio for a single branch, and both perform nearly as well when two branches are relatively lengthened. When a single branch is lengthened, performance (accurate retrieval of the known topology) degrades slowly as relative branch length increases thereafter; Bayesian inference with gamma correction for ASRV performs best among these alternatives. When two branches are relatively lengthened, the performance of ML, and of ASRV-uncorrected Bayesian inference, falls off much more rapidly, whereas in our simulations ASRV-corrected Bayesian inference was more robust than ML. These performance characteristics have been demonstrated only for protein-sequence datasets of the size, length, composition, divergence and tree shape we examined, and for these implementations of ML (PROML) and Bayesian inference (MrBayes). Applicability to larger, longer, and more divergent protein-sequence datasets, to more-diverse tree shapes, and to different implementations seems highly probable, although further nuance will doubtlessly emerge, and scope may remain for further optimization.

### Model violation

Both the mammalian mitochondrial (mtmam) and JTT models embody empirical probabilistic models of amino acid substitution. Codon usage is highly skewed in mitochondrial genomes compared with the cognate nucleocytoplasmic components [50], and the amino acid transition probabilities in mtmam differ correspondingly from those in JTT. Nonetheless, for the datasets we examined, both ML and Bayesian inference perform well, at biologically reasonable ratios of branch-length difference, even when the JTT model is used to infer trees from protein datasets evolved under mtmam (Figures 7, 8, 9, 10). With one exception, the first inaccurately reconstructed trees were observed at the 20-fold ratio (Table 2). Model violation increased the inaccuracy of reconstruction (as measured by the total number of inaccurate trees over the eight branch-length ratios) by 8 to 39% (mean 18%) in the case of one differentially extended branch, and by -6 to 34% (mean 13%) where two branches are lengthened (Table 2). In the former case, the total burden of these inaccuracies was 16% and 18% as assessed by symmetric and edit distances respectively. The effect of model violation on accuracy for trees with two differentially lengthened branches was more variable; little change (or even a reduction in burden) was observed for ML and Bayesian inference without ASRV correction, whereas violating the model greatly decreased the accuracy of reconstruction by gamma-corrected Bayesian inference. Nonetheless, even at this reduced accuracy, gamma-corrected Bayesian inference performed much more-accurately than either ML or uncorrected Bayesian inference at branch-length ratios of 20-fold and greater.

Particularly in simulations where a single branch was differentially lengthened (Table 2), using the wrong model of sequence change sometimes improved some aspects of performance. Thus with ML inference, inference under the wrong model increased both the total number of inaccurate trees and the bipartition burden over the 8 branch-length ratios (400 trees), but each inaccurate tree was, on average, slightly less inaccurate (as assessed by symmetric distance) than those inferred under the correct model of sequence change. The same phenomenon was observed with Bayesian inference using gamma ASRV correction and an exponential distribution of prior probability over branch lengths. With Bayesian inference uncorrected for ASRV, both the total bipartition burden, and the mean inaccuracy of inaccurate trees as assessed by symmetric distance, were lessened under the wrong model. In simulations with two long branches as well (Table 2), we observed that with ML inference, model violation reduces the number of inaccurate trees and the burden of edits required to generate them, although the latter was not seen when using symmetric distance as the metric. Others have reported situations in which using the wrong model improves the performance of ML ([51-53] and pp. 272–274 of [2]). Some of these cases appear to result from the specific placement of long branches in the "anti-Felsenstein zone", where biased estimation can increase the efficiency of finding the correct topology [52,54]. However, this does not explain our results, as we separated the long branches from each other.

The degree of insensitivity to model violation we observe for gamma-corrected ML and Bayesian inference goes some way toward mitigating possible concern (see above under *Support for subtrees*) that the relative performance of these approaches as reported herein might, in part, reflect their differential sensitivity to sub-optimality in the models used.

### Measures of tree comparison

Our results (Figures 2, 3, 4, 5, Figures 7, 8, 9, 10 and Table 2) illustrate how Robinson-Foulds symmetric distance and edit distance provide non-identical, complementary views of topological incongruence. The former metric enumerates the number of internal nodes that must be collapsed to make two topologies identical, whereas the latter counts the number of break-and-reanneal operations needed to convert one topology into another. The scores are identical if all incongruent subtrees can be reconciled by collapse through, or transfer across, a single internal node, but diverge from each other to the extent that incongruent subtrees are positioned more distantly (*i.e. *across more internal nodes) from each other. Our results also illustrate the difference in dynamic range offered by these metrics, while simulation studies [17,55,56] indicate their differential sensitivity to overall tree shape and/or local topology. Other tree-comparison metrics are available and may offer advantages, *e.g. *in distinguishing transformations that affect large numbers of termini from those that affect small numbers of termini, in robustness against displacement of particular termini, or in application to very large trees [57-59].

## Conclusions

Bayesian inference can be as robust as ML against relative branch-length differences of 20-fold or greater in inference of correct topologies from protein-sequence data, although details depend on the number of relatively long branches, the presence or absence of an effective correction for ASRV, and (presumably) other factors. One might doubt that sequences so dissimilar as to produce a 20-fold (or more) difference in branch lengths could be believably recognised as homologous, or reliably aligned. Bayesian inference can also be as robust as ML to violation of the model of amino acid transition probability. For empirical protein-sequence data that might reasonably be encountered in biological research, then, both gamma-corrected ML and gamma-corrected Bayesian inference perform well in recovering the correct topology. As Bayesian inference is typically very much faster than even a single ML run, not to mention than *e.g. *100 or 1000 replicate runs required to estimate bootstrap proportions, ASRV-corrected Bayesian inference must be seen as an important alternative for statistically based phylogenetic analysis of protein-sequence data when computational resources are limiting. It appears that the interpretation of bootstrap proportions and posterior probabilities being developed for nucleotide sequences will apply as well to protein sequences.

Our interest in lateral genetic transfer (LGT) [7-9] led us to investigate different measures with which to characterise topological difference among trees. Whether LGT tends to occur primarily among closely related lineages, or alternatively whether the frequency of transfer depends more critically on some other factor (oligonucleotide frequency, common environment) – or indeed is purely random – remains an open question. Attention has recently been focused on hypotheses that accord to close-range LGT the central role in metabolic and physiological innovation [60] and in shaping organismal phylogeny [61]. A statistic that captures both the number of transfer events (as does edit distance), and the topological breadth of transfer (as does symmetric distance), would thus be valuable in elucidating the pattern and significance of LGT. For such a statistic to be meaningful in a biological context, it must be sensitive to the annotation (specific phyletic value) of the subtrees involved. Implementation of this, and of a broader range of tree-comparison metrics, in platform-independent software should be a matter of some urgency.

## Methods

### Simulated data

Simulated data were evolved using the "evolver" program within PAML version 3.13a [62,63]. First, we generated random trees, each with 7 species (sequences), using the settings birth rate 0.2, death rate 0.2, sampling fraction 0.5, and mutation rate 0.5. In one set of runs, 8 additional trees were then produced, in which 1 of these 7 branches (selected at random) was progressively lengthened to be longer than the others by the factors 5, 10, 20, 30, 40, 50, 60 and 70. In a second set of runs, 8 other trees were produced, in which 2 branches were progressively lengthened by these same factors (each long branch in a given tree was extended by the same factor). The branches to be lengthened were selected to be as distant from each other as possible in the tree; for a strictly bifurcating tree with seven termini (leaves), this means that it would have a Robinson-Foulds symmetric distance ([64]; see below) of 8 if its long branches were forced to become adjacent. For the trees with 1 or 2 branches differentially lengthened 70-fold, branch lengths were reduced proportionally (very slightly) to maintain all absolute values less than 10, so as not to exceed bounds set on Bayesian prior distributions (below).

Protein data sets were then generated on each of the 16 trees with differentially lengthened branches, using the "evolver" program in PAML. On each tree we evolved 100 replicate protein datasets under the JTT model of sequence change [65], with among-sites rate variation (ASRV) modelled as an 8-category discrete approximation to a gamma distribution with alpha (shape) parameter 0.5. In a second set of runs, we similarly evolved 100 datasets under the mtmam [62,63] model, originally named REV [66], estimated from a set of mammalian mitochondrial proteins. Each protein dataset was of initial length 1000 amino acids. From each of the 32 sets of 100 replicate protein datasets, we then selected 50 replicate protein datasets at random for further analysis.

#### Maximum likelihood inference

All maximum-likelihood (ML) trees were inferred using PROML version 3.6a3 in Felsenstein's PHYLIP package [34] implemented on an 8-processor SGI Origin 2100 under IRIX, a 128-processor SUN Netra-1 cluster under Linux, and a 16-processor IBM p690 Regatta under AIX. In all ML inference we assumed the JTT model of sequence change, randomized (jumbled) the order of sequence addition, used global rearrangements, and selected the "not rough" analysis option in PROML. More information on these settings is available online [34]. We assumed an 8-category discrete approximation to a gamma distribution, with values for the gamma shape parameter estimated separately for each dataset using Tree-Puzzle [67], but frequencies for each category estimated by PROML; rates were assumed to be uncorrelated at adjacent sites. For both empirical and simulated data, models and parameter values were selected to facilitate, as much as possible, a fair comparison of ML and Bayesian approaches. Some details of ML inference differed for empirical *vs *simuated data. Here we present methods for the simulated data; methods specific to the empirical data are given below.

For simulated data, we report results from both (1) single ML inference runs based on each of the 50 replicate protein datasets at each branch-length ratio increment, and (2) bootstrapping (*N *= 10) each of the 50 replicate datasets, as described in the preceding paragraph.

#### Bayesian inference

Bayesian inference (B) was carried out using MRBAYES version 2.01 [31] implemented on a 16-processor IBM p690 Regatta under AIX, and on a 508-processor Compaq ES45 cluster under Linux. (Version 3.0 of MRBAYES was not used because, at the time these analyses were carried out, no documentation was available on how to force the shape parameter to remain fixed after initialization). Priors were defined over the branch-length interval (0.0,10.0), and the JTT model of sequence change was assumed (or known to be correct) for all analyses. For empirical data, trees were inferred using two models of sequence change: JTT, and a variant ("equalin", EQ) of the F81 model of Felsenstein [1]. ASRV was modeled as an 8-category gamma distribution, and the shape parameter was optimized by MRBAYES. The prior distribution on branch lengths was assumed to be uniform, and following initial trials (data not shown) the Markov chain temperature was set to 0.2000. For each dataset, 8 Markov chains were propagated for 30,000 generations each and sampled every 100 generations. As preliminary analyses showed convergence within a few thousand generations, burn-in was conservatively set at 10,000 generations. Posterior probabilities were obtained using *allcompat *(*i.e.*, extended 50% majority-rule consensus) among these sampled trees.

For simulated data, we examined three models of different complexities: (1) a uniform prior distribution over branch lengths, and a single rate category; (2) a uniform prior, and an 8-category gamma model of ASRV; and (3) an exponential prior, and an 8-category gamma. The gamma shape parameter was, as above, estimated using Tree-Puzzle, and was fixed (*i.e. *did not merely serve to initialize estimation by MRBAYES). In runs where an exponential prior was used, the value of the exponent was estimated from the simulated data, and differed according to sequence-change model: under JTT, 0.10 for datasets with both one and two long branches, and under mtmam, 1.04 for one long branch, and 0.60 for two long branches.

#### Comparing topologies and subtree reliabilities

For trees inferred from the 16 sets of simulated data (1 or 2 long branches, 8 ratios of branch-length difference), topologies were compared against that of the (known) tree on which the data had been evolved. For this we employed two metrics: (1) the minimum number of break-and-reanneal edits required to convert one tree into the other. This metric goes under various names, including *subtree prune and regraft distance *[33]; we refer to it simply as *edit distance*; and (2) the Robinson-Foulds symmetric distance [64] as implemented in TREEDIST in the PHYLIP package [34]. The values of these metrics were not normalized (cf. [68]) because all simulated trees have the same number of internal edges. Subtree support was assessed as bootstrap proportion (BP) for ML, and as posterior probability (PP) for Bayesian inference.

### Empirical data

Methods and procedures followed those for simulated data (above), except as described subsequently here.

Aligned protein sequence datasets (see Additional file 1) were obtained from Dr Nick Goldman (EBI). We selected 21 datasets (240 sequences in total, mean 11.4 sequences per dataset), requiring each to be of interestingly large size (minimum 8 sequences) but not too large for analysis by bootstrapped protein likelihood, given the computational resources available to us (maximum 16 sequences). These were reformatted for further analysis, assigning new designators to anonymize individual sequences and to avoid the use of characters that are not supported within the rule sets of the software programs we used ("illegal characters").

For empirical data, we inferred ML trees in two ways: (1) using a user-defined hidden Markov model (HMM) with 8 categories, each set to 12.5% of sites, and with rates in each category estimated using Tree-Puzzle version 5.0 [67]; and (2) assuming an 8-category discrete approximation to a gamma distribution, with values for the gamma shape parameter estimated using Tree-Puzzle (but frequencies for each category estimated by PROML), rates at adjacent sites assumed to be uncorrelated, and the among-sites rate variation (ASRV) gamma-shape parameter estimated for each dataset using Tree-Puzzle. In all our use of Tree-Puzzle, we assumed 8 rate categories (each covering one-eighth of the aligned positions) and JTT. For simulated data, ML trees were inferred using only the 8-category discrete approximation to a gamma distribution.

ML analyses were bootstrapped (*N *= 100 for the 21 empirical data sets, *N *= 10 for the 1600 simulated datasets) with preservation of rate-class information as described in the SEQBOOT documentation. Nonparametric bootstrap proportions (BPs) were computed under extended majority rule consensus using CONSENSE. Both SEQBOOT and CONSENSE are in PHYLIP [34].

For the 21 sets of empirical data, no "true" tree is available. Topologies resulting from the different inference approaches and models (ML-JTT-HMM, ML-JTT-gamma, B-JTT, B-EQ) were therefore compared amongst themselves, using edit distance (determined manually) as the comparison metric. Topologies of the bootstrap ML consensus trees were compared as well, although as consensus trees they do not necessarily reflect most-likely topologies.

### Availability of data

The 21 empirical protein-sequence datasets from Dr Nick Goldman, and our simulated datasets with one or two long branches, are available for download at [69].

## Authors' contributions

JCM was responsible for data simulation and analysis. TJH was responsible for high-performance computing, and generated the figures. MAR initiated and supervised the project, and wrote the manuscript.

## Supplementary Material

Additional File 1 **Description of 21 empirical datasets **This PDF file contains information on each of the 21 empirical datasets provided by Dr Nick Goldman, including: number of sequences, GenBank ID (gi number) of first sequence in dataset, key words from description line of first sequence, PAUP* parsimony score of dataset, number of internal nodes, and number of zero-length internal edges observed with PROML to have support in three non-overlapping intervals: P < 0.01, P < 0.05 but not P < 0.01, and worse than P < 0.05. Click here for file
