# Supplementary material for: Bayesian and maximum likelihood phylogenetic analyses of protein sequence data under relative branch-length differences and model violation
Source: BMC Evol Biol. 2005 Jan 28;5:8. doi: 10.1186/1471-2148-5-8 (PMC549035; doi:10.1186/1471-2148-5-8)
Supplement: Additional File 1 — Description of 21 empirical datasets This PDF file contains information on each of the 21 empirical datasets provided by Dr Nick Goldman, including: number of sequences, GenBank ID (gi number) of first sequence in dataset, key words from description line of first sequence, PAUP* parsimony score of dataset, number of internal nodes, and number of zero-length internal edges observed with PROML to have support in three non-overlapping intervals: P < 0.01, P < 0.05 but not P < 0.01, and worse than P < 0.05. [file 1471-2148-5-8-S1.pdf]

		First sequence is:		PAUP*		PROML gamma<br>* = significantly positive, P < 0.05<br>** = significantly positive, P < 0.01		
Goldman dataset	# of sequences	GI	Description	Parsimony score	# of internal nodes	# of zero-length branches (**)	# of zero-length branches (*)	# of zero-length branches
1	8	170111	Ferredoxin-NADP oxidoreductase	641	5	0	0	0
2	8	120283	Flavodoxin	444	5	1	0	1
3	11	15804393	Porphobilinogen deaminase	727	8	0	2	0
4	8	4504669	Interleukin 4	188	5	1	0	0
5	9	124154	Trypsin inhibitor	277	6	0	1	1
6	9	139836	Xylose isomerase	483	6	1	0	0
7	10	3023284	alpha-Amylase	505	7	0	1	2
8	14	26249817	Malate dehydrogenase	840	11	1	0	1
9	13	20141091	Neurotoxin 2 precursor	136	10	4	0	2
10	10	15595715	Cytochrome c-551 precursor	225	7	2	1	1
11	13	115686	Chloramphenicol acetyltransferase III	792	10	1	0	0
12	16	30585005	*Homo sapiens* interleukin 8	324	13	4	0	0
13	13	118985	Dihydrofolate reductase	248	10	3	1	1
14	11	229728	Chain A, Cytochrome	323	8	0	0	2
15	14	128239	Nitrogenase molybdenum-iron protein alpha chain	1354	11	0	0	4
16	12	209486	Tumor necrosis factor precursor	188	9	2	1	0
17	10	230568	Chain A, Hemerythrin (Met)	280	7	2	0	0
18	15	230780	Chain A, Thymidylate Synthase	1034	12	0	0	1
19	16	23103003	Chain A, Dihydrolipoamide Dehydrogenase	1866	13	2	0	0

20	9	5107783	Cytosolic malate dehydrogenase	468	6	1	0	0
21	11	998767	Keratinase	946	8	0	1	0

PROML no gamma

# of zero-length branches (**)	# of zero-length branches (*)	# of zero-length branches	Degree of edit disagreement between Bayesian & ML approaches
0	0	0	None (0 steps)
2	0	0	Minor (1-2 steps)
0	1	0	None (0 steps)
1	0	0	None (0 steps)
0	0	1	Major (>2 steps)
1	0	0	None (0 steps)
0	1	2	Minor (1-2 steps)
0	1	0	Major (>2 steps)
4	0	2	Minor (1-2 steps)
3	0	2	Minor (1-2 steps)
0	0	0	Minor (1-2 steps)
4	0	0	None (0 steps)
4	0	1	None (0 steps)
0	1	0	None (0 steps)
0	1	1	Major (>2 steps)
2	0	0	Major (>2 steps)
0	0	0	Minor (1-2 steps)
0	0	1	Minor (1-2 steps)
1	0	0	Minor (1-2 steps)

1	0	0	Minor (1-2 steps)
0	1	0	Minor (1-2 steps)
